# Epigenetics and Female Reproductive Aging

**DOI:** 10.3389/fendo.2019.00473

**Published:** 2019-08-29

**Authors:** Isaac J. Chamani, David L. Keefe

**Affiliations:** ^1^NYU School of Medicine, New York, NY, United States; ^2^Department of Obstetrics and Gynecology, New York University School of Medicine, New York, NY, United States

**Keywords:** DNA, infertility, aging, reproductive, epigenetics

## Abstract

With more women than ever waiting until a more advanced age to have children, there exists a newfound urgency to identify the various implications aging has on human reproduction, and understand the disrupted biological processes that result in these changes. In this review, we focus on one recent area of study: the age related epigenetic changes that have been found in female reproductive organs, and the effect these changes may contribute to reproductive outcomes.

## Background

As a result of many social and economic factors, increasing numbers of women are delaying childbirth until a more advanced age (1). Increasing maternal age has resulted in well documented decreases in fertility (2, 3), and increases in oocyte aneuploidy (4), and pregnancy complications (5–7). While these risks are clear, the underlying physiological processes that result in these outcomes are much less well defined. Proposed mechanisms involve neuroendocrine deficiency (8, 9), oocyte chromosome telomere length, meiosis abnormalities (10, 11), mitochondrial dysfunction (12, 13), as well as epigenetic modifications. Here, we discuss the epigenetic alterations associated with aging in female reproduction, and their effects on female reproduction.

## Epigenetics: A General Overview

Epigenetics are heritable covalent modifications to DNA bases and chromatin proteins that do not alter the actual base pair sequence, but rather enhance or repress its transcription, by affecting chromatin structure and transcription factor binding (14). Epigenetics are essential to normal development and functioning (15, 16), and play an integral role in both normal cellular function and disease (17). Epigenetic modifications include DNA methylation, histone modifications (18), and chromatin remodeling (19).

DNA methylation (in mammals) predominantly involves adding a methyl group [S-adenosylmethionine (SAM)] to the fifth carbon position of cytosine bases in CpG dinucleotides—a cytosine followed immediately by guanine (20). DNA methyltransferase (DNMT) is the group of enzymes responsible for adding methyl groups to DNA. DNA methylation generally represses transcription of the gene, by condensing the chromatin structure and preventing transcription factors from binding, as well as by coordinating with HDAC's to decrease histone acetylation, another repressive factor (18). During cellular differentiation, however, methylation of 3′ CpG islands has been shown to *activate* transcription (21). Methyl groups can be removed from DNA—demethylation—actively or passively. Active demethylation occurs via the ten-eleven translocation (TET) family of enzymes, while passive demethylation occurs during DNA replication by the lack-of-methylation of newly formed daughter strands (thereby diluting the methylation present) (22–24).

Histones are octamers comprised of two copies of each of four basic building blocks—H2A, H2B, H3, and H4—that are wrapped in DNA forming a nucleosome. Several nucleosomes further wrap together and are stabilized by H1 linker histones (25). Histone modifications come in many forms: acetylation, methylation (mono, di, tri), phosphorylation, ubiquination, and sumoylation (26). Histone acetylation generally promotes transcription by neutralizing the histone's positive charge and thereby reducing its attraction to the negatively charged DNA, allowing the DNA to open (27). Histone acetylation occurs via the histone acetyltransferase enzyme (HAT), and deacetylation occurs via the histone deactylase enzyme (HDAC). Histone methylation can either promote or inhibit transcription depending on the methylation site (28, 29), and has also been shown to influence DNA methylation activity, and vice versa (30). Histone methylation occurs via histone methyl transferase (HMT), while demethylation occurs via lysine demethylase (KDM) (31). Histone phosphorylation is an important regulator of gene transcription, and mitotic chromatin condensation (32). Phosphorylation occurs on serine, threonine, and tyrosine residues via various regulatory kinases. The mechanism by which phosphorylation achieves its effects is thought to be via altered DNA surface binding affinity (26). Histone ubiquitination involves adding a ubiquitin group—a small regulatory protein—onto histones H2A or H2B. Ubiquitination requires three enzymes, E1, E2, E3, in a sequential process, and can result in transcription repression or expression, depending on the location of ubiquination (33). Finally, sumoylation is the addition of a SUMO (small ubiquitin-like modifiers) group to histones or transcription factors, via enzymes E1, E2, E3, and generally resulting in transcription repression (34).

Another very important regulator of cellular function is non-histone protein methylation. These are post-translational modifications that occur on proteins and alter their function. Methyl groups can be added onto lysine or arginine residues of specific proteins. The function of these modifications is not yet completely understood, but they are thought to affect DNA structure and function, RNA and protein synthesis and metabolism, and the cell cycle and apoptosis (35).

## Epigenetic Changes in Female Reproductive Aging

Changes in epigenetics, and epigenetic related enzymes, in oocytes of females of advanced age have been reported to include alterations to DNMT levels, DNA methylation levels, and histone acetylation and methylation patterns (Table 1).

**Table 1:** Epigenetics, their biological function, enzymes, and changes in oocytes with advanced age.

**Epigenetic modification**	**Biological function**	**Enzymes**	**Changes in oocytes with aging**
DNA methylation	↓ transcription 3′ methylation in differentiating cells ↑ transcription	DNMT–methylationTET–demethylation	↓ DNMT's and methylation in MII oocytes and preimplantation mouse embryos (36) ↑ demethylation enzymes and intermediates in mouse oocytes (37) more extreme methylation pattern in human granulosa cells (38)
Histone acetylation	↑ transcription	HAT–acetylationHDAC–deacetylation	↓ in GV mouse oocytes (39) ↑ in MII mouse oocytes ↑ in MI and MII human oocytes (40)
Histone methylation	↑/↓ transcription (location dependent)	HMT–methylationKDM–demethylation	↓ in GV mouse oocytes (41) ↑ di-methylation in MII mouse oocyets (42)
Histone phosphorylation	↑/↓ transcription/chromatin condensation	Kinases	–
Histone ubiquination	↑/↓ transcription (location dependent)	E1, E2, E3	–
Histone sumoylation	↓ transcription	E1, E2, E3	–

### DNMT Alterations in Reproductive Aging

DNA methyltransferase is a group of enzymes responsible for adding methyl groups to DNA (43). There are currently five DNMT's known to function in mammals, each with a slightly different function: DNMT1, DNMT2, DNMT3a, DNMT3b, and DNMT3L. There also are two types of DNA methylation that can occur: maintenance and *de novo*. Methylation maintenance occurs after semi-conservative DNA replication, where the newly synthesized, semi-un-methylated daughter strand, is methylated to the same pattern as the parent template strand. The new hemi-methylated DNA thus becomes fully methylated. Methylation maintenance is the role of DNMT1 (20). On the other hand, when double stranded un-methylated DNA is methylated for the first time, it is known as *de novo* methylation. This process occurs via DNMT3a and DNMT3b (each has slightly different substrate preference) (44). DNMT3L does not itself methylate DNA, but rather it helps facilitate DNMT3a, and DNMT3b activity (45, 46). DNMT2 has a separate function in methylation of transfer RNA (47).

During normal development, as an oocyte develops from primordial, to primary, to secondary follicle, and then from the germinal vesicle (GV) through MII and beyond, the levels of the various types of DNMT's are in flux. DNMT1 is first expressed in the secondary follicle stage, and continues through the zygotic stage and beyond. DNMT3a is expressed beginning from the primordial stage, and DNMT3b is expressed from the primary follicle stage. DNMT2a is not detectable at any stage. DNMT3L is detected in preimplantation embryos (48). The cellular location (cytoplasmic vs. nucleic) in which the enzymes are located also changes with the different stages (49). The levels of DNMT 3a, 3b, and 3L in developing oocytes have been shown to correlate with their levels of growth and DNA methylation, associating them with a unique role in oocyte maturation (50). Furthermore, targeted gene deletions of DNMT3a or DNMT3L result in similar outcomes—embryonic death due to a failure of imprinting (epigenetic silencing of maternal or paternal DNA, that results in only one chromosome being expressed for a certain trait) caused by inadequate methylation—pointing to the essential role these enzymes play (51, 52). Interestingly, oocytes lacking DNMT3b do not show severe abnormality, indicating it plays a secondary role in development (53).

With aging, this pattern of DNMT regulation is altered. Experiments comparing MII oocytes from aged mice (42–45 weeks) to young mice (5–6 weeks) found altered levels of transcription of genes involved in establishing and maintaining DNA methylation, including DNMT1, DNMT3L, and higher levels of DNMT3b transcription (54). Similarly, a study using even older mice (66 weeks old) found reductions in transcription levels of DNMT3a (55). In another study on human oocytes from aged women, genes involved in cell cycle checkpoint and DNA damage repair and transcription showed decreased transcription (56).

Comparisons of DNMT1, DNMT3a, DNMT3b, and DNMT3L levels in MII oocytes and pre-implantation embryos (2 cell, 4 cell, 8 cell, and morula) showed a significant decrease in old (35–40 weeks) compared to young mice (6–8 weeks) (36). This decrease in turn reflects a broader decrease in DNA methylation found with aging in oocytes.

### DNA Methylation Alterations in Reproductive Aging

DNA methylation in germ cells and early embryos is dynamic, and plays a critical role in development and growth throughout life (57). Somatic cells have stable and highly methylated DNA, which regulates their gene expression and allows them to carry out their tissue-specific functions. While mature oocytes and sperm have similarly high levels of methylation (20), they undergo dynamic changes throughout their development.

Early on, during the primordial stage, mouse germ cells undergo genome wide demethylation. Oocytes then enter meiotic arrest, and undergo remethylation only after birth, during oocyte growth from primary to secondary follicles (58). Male germ cells replicate throughout the life of the male, and enter meiosis in the adult male. Remethylation in male germ cells occurs prenatally at the pro-spermatagonia stage.

Post fertilization, the maternal and paternal chromosomes are physically separate, and undergo different methylation changes. The paternal genome is actively-demethylated before DNA replication begins, while the maternal genome is demethylated passively (59–61). By the blastocyst stage, near the time of implantation, both genomes have again been remethylated (58). This cycle of demethylation and remethylation is important for removing parental epigenetic modifications to the germ cell genome, and for establishing totipotency in the new embryo (15, 58).

With maternal aging, these patterns change. By measuring 5-MeC fluorescence intensity in MII oocytes and preimplantation embryos (2-cell, 4-cell, 8-cell, and morula), researchers showed that the level of DNA methylation in older mice (35–40 weeks) was significantly lower than that of younger mice (6–8 weeks). Interestingly, there was no significant difference in DNA methylation in blastocysts (36). The researchers suggested that this may be attributed to the *de novo* methylation which occurs via DNMT3a and DNMT3b prior to implantation. Other researchers similarly showed that in cases of advanced maternal age, embryos capable of developing to mid-gestation seem to undergo normal acquisition and maintenance of DNA methylation patterning (62).

Other research investigated the levels of DNA methylation and related gene transcription in human granulosa cells. Researchers looked at genomic DNA methylation patterns in granulosa cells of young (mean age of 26) and older (mean age of 40) women. The younger group responded robustly to ovarian stimulation during assisted reproductive technology (ART), with a mean of 25 oocytes retrieved, while the older group responded poorly with a mean of <4 oocytes retrieved. DNA methylome patterns between the two groups were compared using Methylated DNA Capture followed by Next Generation Sequencing (MethylCap-seq), as well as by Reduced Representation Bisulfate Sequencing (RRBS). Their results revealed a more nuanced methylation change with aging. Regions of DNA that are more highly methylated DNA in younger females, showed increased levels of methylation with aging, and areas that were poorly methylated in younger females, showed decreased methylation with aging. With aging, then, the DNA methylation pattern shifts farther to both extremes. The researchers then sought to assess the effect this methylation difference might have on gene expression. They found 3,397 genes that were differentially expressed between the two groups, of which 1,809 were downregulated in the older group, and many of which are related to ovarian function [e.g., anti-mullerian hormone (AMH)] (38).

The flip side of DNA methylation is DNA demethylation. Intermediates of the demethylation cascade in mouse oocytes, including levels of TET enzymes as well as modified cytosine's resulting from demethylation (5-hydroxymethylcytosine, 5-formylcytosine, 5-carboxylcytosine), have been found to be increased with advanced maternal age, signaling that the low DNA methylation levels observed with advanced age may result from decreased methylation, as well as increased demethylation. Furthermore, the researchers found that chemically induced accelerated aging results in different levels of demethylation-pathway-intermediates than what is found in normal aging (37). This can potentially be useful in distinguishing between natural and accelerated aging, and perhaps more importantly evaluating the pace of a woman's reproductive aging and her estimated reproductive longevity.

The consequences of altered DNA methylation in advanced maternal age was recently studied, and found to result in decreased expression many important genes. Researchers used single embryo RNA-seq to examine the genetic expression of human blastocysts. They found that with increasing maternal age, there is decreased expression in over 800 genes, including many genes that are critical for cell cycle control and meiotic chromosomal segregation, and are potential causes of aneuploidy with aging (63). This, once again, highlights the important role epigenetics plays in normal reproduction.

### Histone Modifications in Reproductive Aging

Acetylation of the N-terminal tail of histones usually occurs on lysine (K) residues, and promotes transcription (29). Histone methylation can occur on either lysine or arginine residues, and can suppress or promote transcription, depending on the location. Methylation of histone H3 K9 is associated with transcription suppression (64), while methylation of H3 K4, or methylation of arginine on H3 or H4, is associated with transcription promotion (28, 65).

Like DNA methylation, histone modifications are in flux during germ cell development, and play a critical role in normal gametogenesis. In mouse oocytes at the GV stage, histone 3 lysine 9 and lysine 14 are acetylated, while on histone 4, lysines 5, 8, 12, and 16 are acetylated (H3K9, H3K14, H4K5, H4K8, H4K12, H4K16). At MI and MII, these histones are deacetylated by HDAC with the exception of H4K8 (66, 67). Histone acetylation patterns were found to be similarly well monitored (albeit to a slightly different pattern) in bovine, porcine, and sheep (68). In contrast to this, histone methylation was found to be relatively stable during oocyte maturation (69).

With maternal aging, again, these patterns shift. Research in mouse GV oocytes showed that aged (10-month-old) mice had lower levels of acetylation at H4K12 (67/81) and H4K16 (55/92) than young (2-month-old) mice (100% for both H4K12 and H4K16) (39). Additionally, and paradoxically, at the MII stage, all oocytes from young mice were found to be completely deacetylated at H4K12, while 40% of oocytes from older mice were acetylated at H4K12 (39). Interestingly, the researchers were able to correct the aging related MII stage acetylation abnormalities by correcting the GV oocytes acetylation pattern using Trichostatin A (TSA), a histone deacetylase inhibitor.

Another study compared MII oocytes from the same mouse (to eliminate genetic interactions) at a young (3 weeks) and old age (10 months), and again found higher levels of H4K12 acetylation in the older population (70). Indeed, research indicates that H4K12 acetylation levels can potentially be used as a biomarker for oocyte quality, yielding potential clinical significance to this finding (71).

These changes can result in oocyte dysfunction and infertility. Research looking at the effect of elevated histone acetylation levels in MII mouse oocytes showed that inhibiting histone deacetylase during meiosis results in a high frequency of oocyte aneuploidy and embryo death. Another study examined the role HDAC's play in meiosis, by culturing mouse oocytes with TSA. The treated oocytes progressed through MI and MII, fertilized, and developed into blastocysts, as well as the control group did, but they subsequently had lower implantation rates, and higher miscarriage rates, than the control group. Karyotype analysis done on one cell zygotes revealed higher rates of hyper- and hypo- ploidy in the TSA treated oocytes, explaining their high rate of failure. The inhibition of histone deacetylation in oocytes led to greater rates of aneuploidy. The researchers then compared the H4K12 acetylation status of aged and young oocytes, and found them to be elevated in the aged group. They postulated that this results in an inability of the chromosome to adopt the proper conformation necessary to undergo precise segregation during meiosis, and thereby explains the increased aneuploidy rates seen with increased maternal age (72).

Another group similarly found that oocyte age correlated with abnormally elevated levels of histone acetylation on H4K12 in human MI and MII oocytes. This in turn was related to a greater propensity for chromosomes to misalign, leading to more segregation errors in older oocytes (40). In this way, the residual elevation in histone acetylation in aged oocytes at MI and MII carries a very real clinical significance.

In a similar vein, HDAC's were discovered to contribute to the stability of microtubules, and the formation of proper kinetochore-microtubule interactions, by deacetylating H4K16 and alpha tubulin, in cells undergoing meiosis. Alpha tubulin acetylation levels are important in the regulation and stability of the spindle apparatus, and similar to histone acetylation, are essential for proper spindle formation and kinetochore functioning (73, 74). HDAC2 knockout mouse oocytes resulted in hyper-acetylation of H4K16, defective chromosome condensation and segregation, and increased rates of aneuploidy (73). HDAC3 knockout oocytes had higher levels of tubulin acetylation, and impaired spindle assembly, chromosomal alignment, kinetochore-microtubule attachments and chromosomal movement, resulting in higher rates of MI arrest, and aneuploidy (75).

Sirtuins are another family of cellular deacetylases and Sirt2 in particular also plays an important role in deacetylation of alpha tubules and histone H4K16, giving it great importance in normal microtubule and kinetochore function. Sirt2 knockout mouse oocytes show spindle defects, and chromosomal disorganization, and were significantly impaired in completing MI and forming a polar body. Aged mouse oocytes were found to have lower levels of Sirt2, which corresponded to greater defects in forming a meiotic spindle, and aligning chromosomes. What's more fascinating is that overexpression of Sirt2 in aged oocytes by experimental manipulation led to normalized levels of H4K16 and alpha tubulin acetylation levels, and decreased rates of meiotic dysfunction (76).

Other work examined the changes to histone methylation in oocytes with age. There have been some identified decreases in methylation in H3K9me3, H3K36me2, H3K79me2, and H4K20me2 in GV oocytes of old mice when compared to young mice (41). Other researchers found that di- and tri- methylation on H3K4 was decreased in GV oocytes of aged (42–44 weeks) as compared to young (6–8 weeks) mice, and that H3K4 di-methylation was increased in MII oocytes of the aged mice. Furthermore, the expression of proteins encoded by the genes containing these histone changes was found to fluctuate with respect to the histone methylation level (42).

These results highlight the fundamental role epigenetics plays in oocyte functioning, and how it may, at least partially, explain the decreased fertility, and increased aneuploidy seen in females of advanced age. They also highlight potential therapeutic interventions that may reduce meiotic error, and improve treatment outcomes.

Histone phosphorylation, ubiquination, and sumoylation in oocytes from older females have not yet been as well examined, but studies looking at gene expression identified a dysregulation in genes related to ubiquination in aged oocytes, suggesting dysfunction with age (77).

## Summary and Conclusion

In summary, the existing work on epigenetics and female reproductive aging sheds light on some important cellular mechanisms, while raising many more questions. Manipulating DNMT or HDAC levels in mouse oocytes leads to morbid outcomes, illustrating the critical role these enzymes, and epigenetics in general, play in normal development. A better understanding of their function in normal development, and how they are affected by aging, may lend valuable insight into reproduction and reproductive aging.

MII oocytes and preimplantation embryos of aged mice showed a decrease in DNMT enzymes and methylation levels. Aged mouse oocytes also showed an increase in demethylation enzymes and demethylation cascade intermediates. In contrast, human granulosa cells showed a more nuanced change in methylation with advanced age, shifting toward either extreme—increased methylation in highly methylated regions, and decreased methylation in poorly methylated regions. Histone acetylation was paradoxically decreased in GV oocytes, but increased in MII oocytes of aged mice and humans as compared to young mice. They were shown to play a critical role in normal meiosis, and were also responsive to corrective measures. Finally, histone methylation is decreased in GV oocytes of old mice.

Little has been published, thus far, on the changes to histone phosphorylation, ubiquination, and sumoylation, or the changes to non-histone protein modifications, in oocytes with aging, and the consequences those changes may carry. Histone sumoylation, for example, plays a critical role in mitotic regulation, and spindle assembly in oocytes (78). Epigenetic modifications disrupting normal histone sumoylation functioning can theoretically have severe deleterious effects. Further research is necessary to better understand these changes, and, more specifically, as they occur in human oocytes.

It is also critical to better understand mechanisms of epigenetic changes with age, and the factors that may mitigate or accelerate them. Can biologic or environmental elements that produce oxidative stress, which has been shown to disrupt cellular epigenetics (79), be a factor in determining reproductive longevity? Would it be possible to impede this process, or even correct it, as has been done experimentally using TSA to correct for abnormal acetylation levels? Finally, can epigenetic modifications in aged human oocytes be used to predict an oocyte's embryonic development potential? These questions are just a few of many important areas for clarification, and carry the potential of improving, and fundamentally changing, the care millions of women around the world need and receive.

## Author Contributions

IC and DK contributed conception and design of the review. IC searched databases to identify relevant papers, and wrote the first draft of the manuscript. Both authors contributed to manuscript revision, read, and approved the submitted version.

### Conflict of Interest Statement

The authors declare that the research was conducted in the absence of any commercial or financial relationships that could be construed as a potential conflict of interest.
